# A Prospective Longitudinal Study of Seasonality in African Students Living in the Greater Washington, D.C. Metropolitan Area

**DOI:** 10.1100/tsw.2007.110

**Published:** 2007-05-01

**Authors:** Alvaro Guzman, Ryszard Zebrak, Kelly J. Rohan, Irshad A. Sumar, Svetlana Savchenko, John W. Stiller, Adela Valadez-Meltzer, Cara Olsen, Manana Lapidus, Joseph J. Soriano, Teodor T. Postolache

**Affiliations:** ^1^ Mood and Anxiety Program, Department of Psychiatry, University of Maryland, MSTF Building, Room 502, 685 West Baltimore Street, Baltimore, MD 21201, USA; ^2^ Residency Training Program, St. Elizabeths Hospital, 2700 Martin Luther King Avenue, Washington, D.C. 20032, USA; ^3^ Psychology Department, University of Vermont, John Dewey Hall, 2 Colchester Avenue, Burlington, VT 05405-0134, USA; ^4^ Springfield Hospital Center, Sykesville Road, Sykesville, MD 21784, USA; ^5^ Department of Preventive Medicine and Biometrics, Uniform Services University of the Health Services, 4301 Jones Bridge Road, Bethesda, MD, USA

**Keywords:** seasonality, African students, migration, weight gain, visual analog scale, United States

## Abstract

We conducted a prospective, longitudinal study of seasonality in a vulnerable population, i.e., African students who migrated to a temperate climate. Consistent with previous cross-sectional studies, we hypothesized lower mood and energy, and higher appetite and weight, in fall/winter than in spring/summer. Four cohorts of African students attending a year-long nursing school program without vacation in Washington, D.C., were assessed monthly for 1 year. Forty-three subjects (mean age = 33.46 ± 6.25), consisting of predominantly females (76.7%), completed the study. The cohorts began their academic program in different seasons (one each in winter, spring, summer, and fall), inherently minimizing confounding influences on seasonality, such as academic and immigration stress, as well as allowing adjustment for an order effect. At each assessment, students completed three 100-mm visual analog scales for mood, energy, and appetite, and were weighed on a digital scale. For each standardized dependent variable, a repeated measure ANOVA was used and, if a significant effect of month was identified, averages for spring/summer and fall/winter were compared using paired ttests. In addition, a mixed model for repeated measures was applied to raw (nonstandardized) data. Body weight was significantly higher in fall/winter than in spring/summer (p < 0.01). No seasonal differences in mood, energy, or appetite were found. Benefiting from certain unique features of our cohorts allowing adjustment for order effects, this is the first study to identify a seasonal variation in body weight with a peak in winter using longitudinal monthly measurements.

### INTRODUCTION

Although humans have created a microclimate (artificial lighting, heating, and air conditioning) that reduces their exposure to changes in seasons, many individuals manifest behavioral changes reminiscent of those present in species exposed to seasonal variations in light and temperature[1]. The term "seasonality" refers to the degree to which an individual's mood, energy, appetite, weight, sleep duration, and social activities change across the seasons[2]. In one of its most severe manifestations, seasonality is manifested as Seasonal Affective Disorder (SAD). The most common form of SAD (winter type) is characterized by recurrent major depressive episodes during fall or winter that remit in the spring and summer. Subsyndromal SAD (S-SAD) is a more prevalent, less severe form of SAD[2]. Features of winter-type SAD as well as subsyndromal SAD commonly include increased appetite, carbohydrate craving, weight gain, hypersomnia, and loss of interest in activities[3,4]. Few studies used longitudinal rather than cross-sectional approaches to study seasonality.

We followed, prospectively and longitudinally, African immigrants studying in the Washington, D.C. metropolitan area, a population that we previously found using a cross-sectional survey in another sample to be somewhat more vulnerable to change in seasons than their African American peers[5]. The present study measured month-to-month changes in self-reported mood, energy, appetite, and actual body weight for 1 year. We hypothesized that in fall and winter, as compared to spring and summer, the African students would gain weight and report increased appetite, decreased energy, and decreased happiness.

### METHODS

#### Participant Recruitment

African students who were attending a nursing school in Washington, D.C., enrolled in a year-long academic program with no vacations, were invited to participate in the study. Four cohorts of students participated in the study. One cohort began its studies in December, another in March, the third in June, and the final cohort in September. Each cohort consisted of two class times (i.e., one morning-time class and one evening class). Completion of the study was defined as having at least 10 out of 12 monthly measures. Participants' residency in the U.S. was relatively short (mean = 5.78 ± 4.53 years).

#### Measures

- Visual analogue scale (VAS) — The VAS method is an acceptable way to measure degree of feelings with good validity[6]. The VAS has also been used in the research of appetite and pain[7]. Each scale consisted of a horizontal line of 100-mm length, divided by perpendicular tics every 10 mm. Three scales were presented on a single page, one each for "happiness", "energy", and "appetite". We used a 100-mm scale because it is convenient, fast, and readily understood by participants[6]. The anchors of our scales were 0 for "most unhappy", "no energy", and "no appetite", and 100 for "happiest", "most energetic", and "biggest appetite", respectively. Participants placed a mark along each VAS, which could be measured with a resolution of 1 mm.
- Body weight — Weight (in pounds) was obtained using a battery-powered scale with digital display (Taylor Precision Products L.P., Las Cruces, NM).

### Procedure

The purpose and terms of participation were presented to groups of students in their classroom. Participants reviewed the informed consent document to be signed, with assurance that the study was confidential and would not have any bearing on their academic outcome. At the outset, every student had the opportunity to use a coded name for study purposes (a combination of letters and/or numbers) in place of his or her real name. Each month, students completed the VAS and their weights were measured. Weight was recorded at the same time of day (i.e., before lunch for students who were sampled in the morning and before dinner for students who were sampled in the evening) throughout the study. The nursing school program lasted exactly 1 year without vacations and all participants were followed monthly for 12 consecutive months.

Every student had an opportunity to withdraw at any time during the study. Participants who missed more than two consecutive assessments were excluded from the analysis. No compensation was provided. The Institutional Review Board (IRB) of the D.C. Department of Mental Health and the administration of the nursing school approved this study.

### Statistical Analysis

Two distinct analyses were performed on: a) standardized data and b) raw data. The dependent variables (weight, appetite, mood, and energy) were aligned according to the “calendar month of the year” (rather than the “month since entering the study”) and z-score transformed within subjects. Repeated measure ANOVAs were performed for each monthly value of each variable. For those variables for which the repeated measure ANOVA was significant ($\alpha = 0.05$), averages of the monthly z-scores for spring/summer and fall/winter intervals were calculated and compared using paired t-tests in Systat 9 for Windows. Spring/summer included the months of March, April, May, June, July, and August, and fall/winter included the months of September, October, November, December, January, and February.

For analysis of nonstandardized (raw) data, we used a mixed model for repeated measures data[8]. For each dependent variable, season was included as a within-subjects factor and cohort (start month) was included as a between-subjects factor. In some models, age was included as a between-subjects confounder, but this did not affect the results appreciably, so those results are not presented. After evaluating several alternatives, a first-order autoregressive structure was assumed for the within-subject correlation. Analyses were performed using the MIXED procedure in SAS(R) 8.02 for Windows.

## RESULTS

### Participant Characteristics

A total of 161 African students consented to the study procedures. However, only 44 participants were able to complete the monthly assessments for 10 months or more. Subsequently, one participant was excluded due to pregnancy. Hence, 43 participants were included in the final analysis. This included 33 females, 9 males, and 1 with no gender reported. Mean age was 33.46 (SD = 6.25) with a range of 21–47 years.

### Analysis of Standardized Data

For z-score transformed body weight, the repeated measures ANOVA was significant, $F(11, 1) = 5.23$, $p < 0.01$ (see Fig. 1). Following up with a paired t-test, we found a highly significant increase in body weight during fall/winter in comparison to spring/summer with $t = 4.495$, $N = 43$, $p < 0.001$, which was concordant with our hypothesis (Figs. 1 and 2). The repeated measure ANOVAs were not significant for appetite, mood, and energy level.

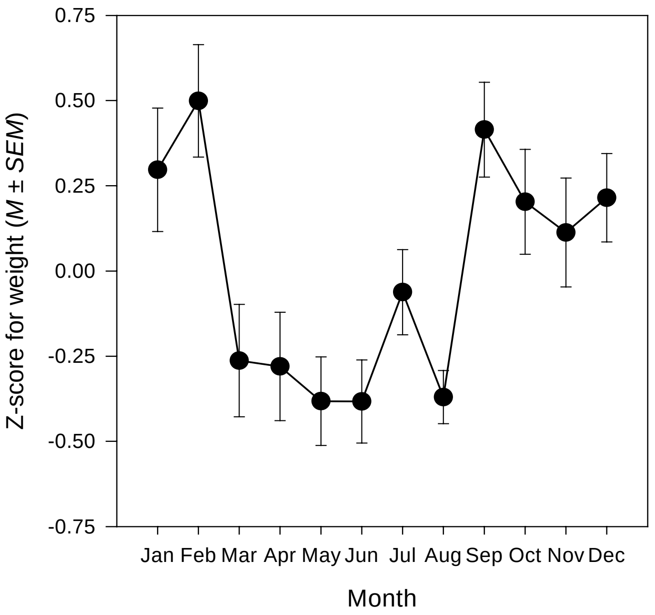

**FIGURE 1.** Seasonal variation in weight. Data in four cohorts are aligned according to the month of the year rather than the month of the study. The data are means and SD of the data representing z-score transformations within each subject. Repeated measure ANOVA was significant at $p < 0.01$.

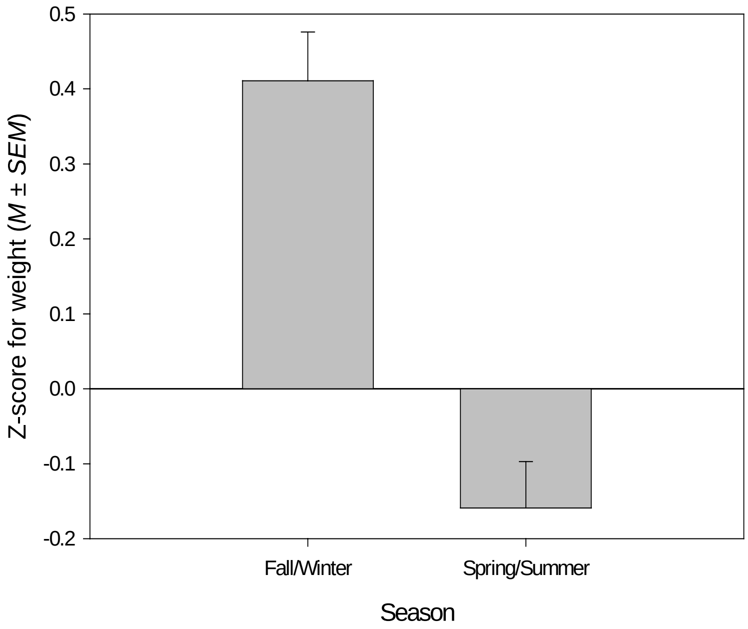

**FIGURE 2.** Averages of fall/winter vs. spring/summer z-scores transformation of weight (within individuals). Paired t-test was significant at $p < 0.001$.

### Analysis of the Nonstandardized (Raw) Data

After adjusting for start month, subjects weighed an average of 3 lbs more in the fall and winter months than in the spring and summer months ($p = 0.0023$). Further adjusting for age yielded an estimated weight difference of 3.8 lbs ($p = 0.0016$). There were no significant seasonal differences with respect to appetite, mood, or energy (Table 1).

**TABLE 1**
**Regression-Adjusted Comparison between Seasons of Raw Data of Each Dependent Variable***

| Dependent Variable | Adjusted Mean, Fall/Winter | Adjusted Mean, Spring/Summer | Difference | *p* Value |
|---|---|---|---|---|
| Weight (lb) | 176.41 | 173.45 | 2.95** | 0.0023 |
| Appetite | 65.29 | 64.61 | 0.68 | 0.7311 |
| Mood | 63.72 | 63.89 | –0.17 | 0.9297 |
| Energy | 59.71 | 59.30 | 0.42 | 0.8475 |

* The values are adjusted for the start month of the cohort. Weight is measured in pounds. Appetite, mood, and energy were measured in millimeters on a 100-mm VAS.

** Significant finding.

### DISCUSSION

Our prospective, longitudinal study of seasonality was unique in several ways: (1) we used an understudied population (i.e., African immigrants), (2) participants had relocated from a warmer climate to a more temperate climate, (3) the study protocol was initiated in a way that controlled for the start month (e.g., familiarity with the environment, academic stress, etc.) as a potential confounder to seasonal effects, and (4) we actually weighed subjects rather than asking about their weight. Another strength of the study was analyzing both z-scores and raw data, each method with its own advantages. The analysis of z-scores minimized the impact on the average of each month of an occasional absence of certain students who represented outliers for any specific variable (e.g., the absence of a very slim student would reduce the average for a specific month). The analysis of raw data employing the mixed model for repeated measures has the advantage of allowing explicit modeling of the within-subjects covariance structure. It is generally inappropriate to assume that repeated assessments on the same subjects are statistically independent. In this data set, it appeared that the correlation between assessments on a subject diminished with increasing time. By modeling this relationship with a first-order autoregressive covariance structure, which assumes that the correlation declines exponentially over time, we were able to fit the data well with only a few parameters, and without the more restrictive assumption of sphericity. Finally, unlike paired t-tests, the mixed model analysis was able to adjust for potential confounding variables, in this case, start month and age. More than half of the subjects included in the analysis (23/43) belonged to the cohort that began in March. Without statistical adjustment, the seasonal patterns for this cohort would have dominated the results. It is important to mention that the results resulting from z-scores and raw data approaches were nearly identical.

To date, there are only a few prospective longitudinal studies of seasonality, two in healthy subjects[9,10], two in indigenous populations compared to newcomers[11,12], and two in mood disorder populations[13,14]. We have identified only one study where weight was measured longitudinally[15].

As expected, the participants had significantly greater body weight in fall/winter than in spring/summer. The objective weight change in this study is consistent with self-reported weight changes in other studies[11,16,17,18]. In contrast to the expected changes in weight, there were no significant seasonal differences in mood, energy, or appetite. These findings differ from other longitudinal studies that have reported both decreased energy and increased depression during winter[10,11,12,19]. The differences between those studies and our findings may be explained by small sample size and/or population differences.

Students' weight gain in winter and fall may be a consequence of the previously described brain serotonin turnover during these seasons[20], given the association of low levels of brain serotonin with decreased satiety[21,22,23]. Independent of this explanation for these weight fluctuations, this finding has several important implications. It suggests that actual weight measurement is a sensitive indicator of seasonality. Also, given the morbidity and mortality associated with weight fluctuations[24,25,26] and the response of subsyndromal SAD of winter type to bright light treatment[27], our results raise an important question: Could light treatment be used to prevent seasonal weight fluctuations, and would this in turn result in better health and longer life?

The results of this study should be viewed in light of several important limitations. The subsample of completers may not be representative of the initial study group, students at this institution, or African students in the U.S. These findings require replication in other African immigrant samples. As a second limitation, we only followed our sample for a 1-year period. Longer study intervals would allow identification of students who reliably experience seasonal behavior changes each year. Third, we did not conduct diagnostic interviews with the students sampled to determine mental health status. Also energy, mood, and appetite were assessed using a relatively crude, self-report measure (the VAS). Measures with stronger reliability and validity would have been preferable; however, we wanted to reduce participant burden and increase compliance with the monthly assessments. It will be important, yet difficult, to attempt to find a similar setting (i.e., a relatively constant schedule for at least 1 year, without vacation), and to use the current approach in the general population and other ethnic or immigrant groups.

In summary, this is the first study to demonstrate, by directly measuring weight on a monthly basis and adjusting for an order effect, that body weight is higher in fall and winter as compared to spring and summer. This may have relevance for evaluating seasonal variation in cardiovascular risk factors and weight reduction programs. The next step would be to measure monthly weight in clinical SAD patients and controls, testing the (not yet confirmed) hypothesis that patients with SAD gain more weight in winter as compared with healthy control subjects. We propose that for monitoring SAD and its response to treatment, a simple weight scale should join the battery of mood rating scales commonly used in the field.

In addition to immigration stress, adaptation to changes in seasons, and especially to winter, may represent an additional physiological and mental challenge for immigrants from warmer climates and lower latitudes. In our opinion, educating immigrants from lower latitudes regarding the potential causes and consequences of these seasonal changes, as well of the availability of affordable and safe treatment modalities for winter type SAD, such as bright light treatment, should parallel ongoing research efforts.

### ACKNOWLEDGMENTS

This study was supported by the Department of Mental Health of the District of Columbia (T. T. Postolache, Principal Investigator).
